# Adapted digital health literacy and health information seeking behavior among lower income groups in Malaysia during the COVID-19 pandemic

**DOI:** 10.3389/fpubh.2022.998272

**Published:** 2022-09-14

**Authors:** Roy Rillera Marzo, Hana W. Jun Chen, Khadijah Abid, Shekhar Chauhan, Mark Mohan Kaggwa, Mohammad Yasir Essar, Jacynta Jayaram, Manah Chandra Changmai, Mohamad Khairuddin bin Adbul Wahab, Indang Ariati Binti Ariffin, Muhammad Najib Bin Mohamad Alwi, Michael G. Head, Yulan Lin

**Affiliations:** ^1^International Medical School, Management and Science University, Kuala Lumpur, Malaysia; ^2^Department of Public Health, The Shaheed Zulfikar Ali Bhutto Institute of Science and Technology, Karachi, Pakistan; ^3^Department of Family and Generations, International Institute for Population Sciences, Mumbai, India; ^4^Department of Psychiatry and Behavioral Neurosciences, McMaster University, Ontario, ON, Canada; ^5^Department of Dentistry, Kabul University of Medical Sciences, Kabul, Afghanistan; ^6^Clinical Informatics Research Unit, Faculty of Medicine, University of Southampton, Southampton, United Kingdom; ^7^Department of Epidemiology and Health Statistics, School of Public Health, Fujian Medical University, Fuzhou, China

**Keywords:** COVID-19, health literacy, digital, health information seeking, lower income

## Abstract

**Background:**

Misinformation has had a negative impact upon the global COVID-19 vaccination program. High-income and middle-income earners typically have better access to technology and health facilities than those in lower-income groups. This creates a rich-poor divide in Digital Health Literacy (DHL), where low-income earners have low DHL resulting in higher COVID-19 vaccine hesitancy. Therefore, this cross-sectional study was undertaken to assess the impact of health information seeking behavior on digital health literacy related to COVID-19 among low-income earners in Selangor, Malaysia.

**Methods:**

A quantitative cross-sectional study was conducted conveniently among 381 individuals from the low-income group in Selangor, Malaysia. The remote data collection (RDC) method was used to gather data. Validated interviewer-rated questionnaires were used to collect data *via* phone call. Respondents included in the study were 18 years and older. A normality of numerical variables were assessed using Shapiro-Wilk test. Univariate analysis of all variables was performed, and results were presented as means, mean ranks, frequencies, and percentages. Mann-Whitney U test or Kruskal Wallis H test was applied for the comparison of DHL and health information seeking behavior with characteristics of the participants. Multivariate linear regression models were applied using DHL as dependent variable and health information seeking behavior as independent factors, adjusting for age, gender, marital status, educational status, employment status, and household income.

**Results:**

The mean age of the study participants was 38.16 ± 14.40 years ranging from 18 to 84 years. The vast majority (94.6%) of participants stated that information seeking regarding COVID-19 was easy or very easy. Around 7 percent of the respondents cited reading information about COVID-19 on the internet as very difficult. The higher mean rank of DHL search, content, reliability, relevance, and privacy was found among participants who were widowed, had primary education, or unemployed. An inverse relationship was found between overall DHL and confidence in the accuracy of the information on the internet regarding COVID-19 (β = −2.01, 95% CI = −2.22 to −1.79).

**Conclusion:**

It is important to provide support to lower-income demographics to assist access to high-quality health information, including less educated, unemployed, and widowed populations. This can improve overall DHL.

## Introduction

The World Health Organization (WHO) defines Digital Health Literacy as the ability to utilize electronic devices to gain, seek, appraise, and understand health information to enhance health outcomes or solve a health issue (1). Recent advancement in technology has made the world more digitalized than before, and thus most populations have access to information about healthcare.

Access to timely and quality information during infectious diseases outbreak is critical to prevent the spread of infection and control the feelings of anxiety. Digital platforms are the main focal points where information exists and spreads (2). Quality and up-to-date information from such platforms about the source of the pandemic, specific health threats, dissemination, mortality, can minimize the risk of infection and public anxiety. However, access to online quality information has been a challenge for vulnerable population such as migrants and the older group. There is a disparity that exists in digital health equity which needs to be highlighted (3, 4).

Social media platforms (Facebook, Twitter, Instagram, etc.) has become a perfect source for health information to flow. There are significant quantities of good and bad public health messaging on social media platforms, which can impact individual and population beliefs and behaviors. In light of the ongoing pandemic, misinformation about the source of the pandemic had become increasingly available on different social media platforms (5). Hence, the pandemic highlighted the negative impacts of false misinformation on all facets of life (6). Misinformation about the source of the Coronavirus disseminated rapidly all across the world that even the WHO coined another word “infodemic,” an overabundance of information and the rapid spread of misleading or fabricated news, images, and videos (7).

Vaccine hesitancy which is one of main global health issues has also taken a surge because of the bulk of misinformation available on social media platforms (8). In studies published, it has shown that the population's decision to vaccinate was influenced by the information on digital platforms (9, 10). Concerns about side effects of the vaccines, rapid development of the vaccines have all contributed to vaccine hesitancy (11, 12). On the other hand, in some countries, the digital platforms have increased public trust on vaccines (13). Thus, it is critical to monitor the digital platforms and make good use of them to help people in their decision making.

Studies have unanimously agreed that COVID-19 has severe health repercussions, including quality of life (14), mental health (15–19), and psychological distress (20–25). Misinformation and vaccine efficacy also impacted the global COVID-19 vaccination program, driving vaccine hesitancy (26–28). However, recommendations from medical professionals' were associated with vaccine acceptance (29, 30). Safety is one of the key population concerns, and in many countries, misinformation has led people to believe that vaccines are not safe, thus increasing hesitancy (31). It is one of the many reasons why the pandemic has not ended.

Malaysia, a Southeast Asian country, has had its own struggles with the pandemic. As of July 14 2022, 4.6 million cases and 35.8 thousand deaths have been reported (32). The country began COVID-19 vaccination in February 2021, and as of 14 July 2022, Malaysia has administered at least 71.5 million doses of COVID vaccines so far, assuming every person needs two doses (33).

Malaysia's population is divided into three categories based on their household income. T20 is also known as the Upper group, which represents the top 20% of the Malaysians; M40, also known as Middle-income, which represent 40% of the Malaysians; and B40%, also known as the Lower-income group, which represents 40% of the Malaysians (34).

High-income and middle-income earners typically have better access to technology and health facilities than the B40 lower-income group (35). Lower-income groups may also have less access to healthcare; there is previous evidence of greater vaccine hesitancy within these demographics (29, 36). Therefore, it is increasingly important to review the engagement of lower income groups in misinformation and identify how best to provide educational support for them using social media and other digital platforms. In addition, it is proven that digital health literacy contributes to better health outcomes (37). This cross-sectional study was undertaken to assess the impact ofhealth information seeking behavior on digital health literacy related to COVID-19 among low-income earners, also known as “B40,” to provide an update for health policymakers on the use of digital health among B40 group and contribute to the improving of their health condition.

## Methods

### Study setting and population

This cross-sectional study was conducted *via* telephone and according to the protocol approved by the Ethics Committee of Management and Science University (Ethics Code: MSU-RMC-02/FR01/09/L1/085). A quantitative cross-sectional study was conducted conveniently among 381 individuals from the low-income group in Selangor, Malaysia. People from lower socioeconomic classes are vulnerable populations negatively affected by the COVID-19 pandemic, thus exacerbating disparities in digital health literacy. According to the Raosoft online sample size calculator (Raosoft, Seattle, WA, US), assuming a 5% margin of error, a 95% confidence level, and a 50% response distribution, the required sample size for this study was 377.

The survey was conducted between 20 September to 3 October 2021 (during the MCO 3.0). The questionnaire was piloted on a sample of 30 to test its validity and reliability, and data obtained from the pilot study were not included in the final analysis. A total of 381/452 (84.3%) participants completed the survey. The remote data collection (RDC) method was used to gather data. Validated interviewer-rated questionnaires were used to collect data *via* phone call. Respondents included in the study were 18 years and older, belonged to the low-income group (B40), living in Selangor. Only one response was allowed per contact number in the telephone survey. We got the list of names and mobile numbers from our university, who adopted the said community.

### Study instruments

This study used a questionnaire that was available in both Bahasa Melayu and English languages. Before questionnaire distribution, a back-to-back translation, content and face validity, and reliability test were done. The questionnaire consisted of 13 items and was divided into three sections. The following data were collected upon the completion of each questionnaire: Section A – sociodemographic profile (6 items), Section B – digital health literacy (5 items from the Digital Health Literacy Instrument (DHLI), adapted from Vaart and Drossaerts, 2017 (37), Section C – health information seeking behavior (2 items, self-developed). The online survey has fulfilled the criteria in the Checklist for Reporting Results of Internet E Surveys (CHERRIES) (2).

### Sociodemographic profile

The sociodemographic characteristics collected for this study were age, gender, marital status, education level, household income and employment status.

#### Digital health literacy

The questions used to assess digital health literacy were adapted from the Development of the Digital Health Literacy Instrument (37). This study adopted five items – one item from every five key dimensions of DHLI, namely, information seeking, adding self-generated content, evaluating reliability, determining relevance, and protecting privacy. The scale measures one's ability to seek, find, understand, and appraise health information from digital resources. This study used the following five key dimensions of DHLI, namely, (1) information searching or using appropriate strategies to look for information (e.g., “When you browse the internet to find information regarding the Coronavirus or related topics, how easy or difficult is it for you to find the exact information?”) (2) adding self-generated content to online-based platforms (e.g., “When typing a message (e.g., on a forum or social media such as Facebook or Twitter) about the coronavirus a related topic. How easy or difficult is it for you to express your opinion, thought, or feelings in writing??”) (3) evaluating the reliability of online information (e.g., “When you search the internet for information on the coronavirus or related topics, how easy or difficult is it for you to decide whether the information is reliable or not?”) (4) determining the relevance of online information (e.g., “When you search the internet for information on the coronavirus or related topics, how easy or difficult is it for you to use the information you found to make decisions about your health (protective measures, hygiene regulations, transmission routes, risks and their prevention?”) and (5) protecting privacy (e.g., “When you post a message about the coronavirus or related topics on a public forum or social media, how often do you share your own private information such as your name or address?”) A total of 5items were asked and it uses a four-point Likert scale: 1 = very difficult, 2 = difficult, or some of the time, 3 = easy, and 4 = very easy.

#### Health information-seeking behavior

This section consisted of 2 self-developed questions to assess health information-seeking behavior. Each item was scored on a 5-point Likert scale. The first question is “*How often do you read information about COVID-19 on the internet*” for which the response options are 5 (at least once a day), 4 (at least once a week), 3 (at least once a month), 2 (less than once a day), and 1 (never). The second question is “*I am confident in the accuracy of the information that I see and read in social media*,” with response options ranging from 5 (strongly agree), 4 (agree), 3 (neutral), 2 (disagree), and 1 (strongly disagree).

### Validity and reliability

A group of expert panels were included such as psychiatrists, clinical psychologists, physicians, pharmacists, and public health experts translated and culturally validated the questionnaire. The set of questions included for Content Validation Index (CVI) calculation was five questions in Section B (digital health literacy) and two questions in Section C (health information seeking behavior). All the questions received an acceptable CVI of more than 70%. The final CVI for both questionnaires calculated was from 88.5 to 97.5%. Other psychometric properties such as face validity and reliability were assessed by conducting a pilot study of 30 subjects. The final face validity index for both questionnaires ranged from 92.5 to 94.7%, and the internal consistency for all the sections was good, with Cronbach's alpha values ranging from 0.87 and 0.94.

### Data analysis

Data were analyzed using Statistical Package for Social Sciences (SPSS) statistical software version 25.0. The normality of numerical variables were assessed using the Shapiro-Wilk test. Univariate analysis of all variables was performed, and results were presented as means, SDs, mean ranks, frequencies, and percentages. Mann-Whitney U test or Kruskal Wallis H test was applied to compare DHL and health information-seeking behavior with the characteristics of the participants. Linear regression was applied by taking overall DHL as the dependent variable and health information-seeking behavior as independent factors. A multivariate linear regression model was derived for overall DHL and health information-seeking behavior after adjusting for age, gender, marital status, educational status, employment status, and household income. A *p*-value of < 0.05 was considered as statistically significant.

## Results

The mean age of the study participants was 38.16 ± 14.40 years ranging from 18 to 84 years, and most participants were of age < 40 years (53.8%). Of 381 participants, 59.3% were females, and 40.7% were males. Most participants were married (55.4%), followed by singles (35.4%). Almost 39.4% of the participants had secondary level education, 56.2% were employed, and 59.3% had household income < RM2,500 per month (B1, ~$560 US dollars).

Table 1 depicts the proportion of respondents who reported digital health literacy and health information-seeking behavior during COVID-19. Almost two-fifths (39.5%) of respondents stated that the information searching/seeking regarding COVID-19 was very easy, and more than half (55.1%) stated that it was easy. Only 5% of the respondents could find information searching/seeking difficult or very difficult. Almost one-fourth of the respondents stated that it was difficult to add self-generating content (26.6%) and to evaluate the reliability (24.6%) of the COVID-19-related digital health literacy. Another one-third (33.9%) find it difficult to protect privacy. More than half of the respondents (55.1%) read information about COVID-19 at least once in a day, and one-third (36.5%) received so at least once a week.

**Table 1:** Digital health literacy and health information seeking behavior of participants (*n* = 301).

**Level of DHL (digital health literacy)**	**Very easy**		**Easy**	**Difficult**	**Very difficult**
Information searching/seeking	119 (39.5)		166 (55.1)	15 (5)	1 (0.3)
Adding self-generated content	68 (22.6)		137 (45.5)	80 (26.6)	16 (5.3)
Evaluating reliability	69 (22.9)		148 (49.2)	74 (24.6)	10 (3.3)
Determining relevance	69 (22.9)		174 (57.8)	49 (16.3)	9 (3)
Protecting privacy	60 (19.9)		106 (35.2)	102 (33.9)	33 (11)
**Health information-seeking behavior**	**Never**		**At least once a day**	**At least once a week**	**At least once a month**
How often do you read information about COVID-19 on the internet?	4 (1.3)		166 (55.1)	110 (36.5)	21 (7)
	**Strongly disagree**	**Disagree**	**Neutral**	**Agree**	**Strongly agree**
**I am confident with the accuracy of the information I read about COVID-19 on social media**.	4 (1.3)	41 (13.6)	102 (33.9)	116 (38.5)	38 (12.6)

Data presented as n (%).

Table 2 compares respondents' characteristics and the overall DHL and its five components by the Mann-Whitney U test or Kruskal Wallis test. Overall, a higher DHL mean rank was found among the participants age > 60 years (mean rank = 261), who had primary education (mean rank = 242.76) and who were not employed (mean rank = 226.03). A statistically significant difference in overall DHL was observed for educational level (*p* = 0.001) and employment status (*p* = 0.001). The higher mean rank of DHL search, content, reliability, relevance, and privacy was found among participants who were age>60 years widows, had primary education, and who were not employed. Statistically significant results were noted for DHL content, reliability, relevance, and privacy by marital status, educational status, and employment status (*p* < 0.05). A statistically significant difference was observed in DHL contents with respect to household income (*p* = 0.001).

**Table 2:** Comparison of participants' characteristics and digital health literacy (*n* = 301).

**Characteristics**	**Overall**	**DHL**	**DHL**	**DHL**	**DHL**	**DHL**
	**DHL**	**search**	**contents**	**reliability**	**relevance**	**privacy**
**Age groups**						
<40 years	168.82	171.16	170.98	166.86	163.68	162.79
40–60 years	210.39	208.14	208.45	211.86	215.99	215.76
>60 years	261.95	255.86	255.41	269.91	270.68	280.57
***p*** **-value**	0.001^*^	0.001^*^	0.001^*^	0.001^*^	0.001^*^	0.001^*^
**Gender**						
Male	195.07	193.60	192.54	200.54	200.93	195.65
Female	188.21	189.21	189.95	184.46	184.19	187.81
***p*** **-value**	0.55	0.68	0.82	0.14	0.12	0.48
**Marital status**						
Single	180.20	179.23	179.56	172.61	171.51	169.47
Married	192.37	193.82	191.90	198.51	197.60	198.28
Divorced	170.57	174.79	173.86	183.93	192.29	198.07
Widowed	263.18	258.62	262.09	252.94	248.26	282.18
Single parent	198.64	187.09	215.18	181.45	214.27	170.14
***p*** **-value**	0.06	0.05	0.04^*^	0.03^*^	0.02^*^	0.001^*^
**Educational level**						
Primary	242.76	238.45	233.94	251.28	251.55	256.01
Secondary	200.64	201.45	203.84	203.75	204.43	207.29
Post-secondary education (pre-university/Diploma)	175.42	167.21	181.87	170.20	168.44	170.28
Tertiary education (Degree/Master)	168.57	183.88	155.11	166.30	167.78	155.78
***p*** **-value**	0.001^*^	0.001^*^	0.001^*^	0.001^*^	0.001^*^	0.001^*^
**Employment status**						
Employed	178.43	181.53	177.40	178.32	183.19	182.71
Not employed	226.03	223.74	226.32	229.40	219.92	224.28
Student	180.89	174.59	183.62	176.56	174.79	170.23
***p*** **-value**	0.001^*^	0.001^*^	0.001^*^	0.001^*^	0.01^*^	0.001^*^
**Household income** per month (Malaysian ringgit)						
< RM2,500 (B1)	197.99	196.22	208.52	193.93	196.53	197.77
RM2,501 – RM3,169 (B2)	176.02	176.40	168.62	179.93	175.15	184.59
RM3,170 – RM3,969 (B3)	180.76	187.44	169.63	200.96	190.34	174.85
RM3,970 – RM4,849 (B4)	192.17	196.07	154.06	189.35	194.35	178.90
***p*** **-value**	0.42	0.49	0.001^*^	0.70	0.45	0.49

Data presented as mean rank.

Mann-Whitney U test/Kruskal Walis test was appli ed.

* Significant at 0.05 level of significance.

*Post-hoc* analysis of all the factors which were significant in Kruskal Walis test is displayed in Table 3.

**Table 3:** *Post-hoc* analysis in Kruskal Walis test.

	**Overall**	**DHL**	**DHL**	**DHL**	**DHL**	**DHL**
	**DHL**	**search**	**contents**	**reliability**	**relevance**	**privacy**
**Age groups**						
<40 years-40–60 years	0.001^*^	0.002^*^	0.003^*^	0.001^*^	0.001^*^	0.001^*^
40–60 years->60 years	0.114	0.127	0.157	0.048^*^	0.062	0.024^*^
<40 years->60 years	0.001^*^	0.001^*^	0.001	0.001^*^	0.001^*^	0.001^*^
**Marital status**						
Married-divorced			0.999	0.999	0.999	0.999
Married-single			0.999	0.26	0.225	0.146
Married-single parent			0.999	0.999	0.999	0.999
Married-widow			0.087	0.408	0.527	0.019^*^
Divorced-single			0.999	0.999	0.999	0.999
Divorced-single parent			0.999	0.999	0.999	0.999
Divorced-widow			0.642	0.999	0.999	0.803
Single-single parent			0.999	0.999	0.999	0.999
Single-widow			0.025^*^	0.031^*^	0.040^*^	0.001^*^
Single parent-widow			0.999	0.999	0.999	0.069
**Educational status**						
Tertiary education-post secondary education	0.999	0.999	0.563	0.999	0.999	0.999
Tertiary education-sSecondary education	0.259	0.999	0.010^*^	0.088	0.091	0.006^*^
Tertiary education-primary education	0.004^*^	0.050^*^	0.001^*^	0.001^*^	0.001^*^	0.001^*^
Post-secondary education-secondary education	0.343	0.038^*^	0.533	0.054	0.026^*^	0.027^*^
Post-secondary education-primary education	0.005^*^	0.001^*^	0.046^*^	0.001^*^	0.001^*^	0.001^*^
Secondary education-primary education	0.19	0.276	0.688	0.073	0.069	0.068
**Income groups**						
B4-B2			0.999			
B4-B3			0.999			
B4-B1			0.026^*^			
B2-B3			0.999			
B2-B1			0.019^*^			
B3-B1			0.276			

* *P* < 0.05.

Table 4 shows the results for means of the health information-seeking behavior by participants' characteristics using the Mann-Whitney *U* test/Kruskal Walis test. Health information-seeking behavior regarding how often the respondents read information about COVID-19 on the internet was significantly associated with age, marital status, educational status, and employment status (*p* < 0.05). Respondents' confidence in the accuracy of the information they read about COVID-19 on social media was found to be significantly associated with age, marital status, educational level, and employment status.

**Table 4:** Comparison of participants' characteristics and health information-seeking behavior (*n* = 301).

**Characteristics**	**Health information-seeking behavior**	
	**How often do you read information about COVID-19 on the internet?**	**I am confident with the accuracy of the information I read about COVID-19 on social media**.
**Age groups**		
≤ 20 years	166.28	229.98
21–25 years	167.79	224.88
>25 years	199.50	178.14
***p*** **-value**	0.027^*^	0.001^*^
**Gender**		
Male	195.38	185.89
Female	187.99	194.50
***p*** **-value**	0.494	0.44
**Marital status**		
Single	164.62	222.93
Married	202.86	173.86
Divorced	182.93	256.93
Widowed	269.38	107.32
Single parent	171.32	215.32
***p*** **-value**	0.001^*^	0.001^*^
**Educational level**		
Primary	268.23	122.97
Secondary	202.53	168.37
Post-secondary education (pre-university/Diploma)	169.53	221.80
Tertiary education (Degree/Master)	160.56	223.74
***p*** **-value**	0.001^*^	0.001^*^
**Employment status**		
Employed	182.59	196.12
Not employed	230.02	147.39
Student	162.65	235.77
***p*** **-value**	0.001^*^	0.001^*^
**Household income**		
< RM2,500 (B1)	200.26	182.82
RM2,501 – RM3,169 (B2)	182.75	196.45
RM3,170 – RM3,969 (B3)	188.09	206.85
RM3,970 – RM4,849 (B4)	155.08	214.54
***p*** **-value**	0.08	0.26

Data presented as mean rank.

Mann-Whitney U test/Kruskal Walis test was applied.

* Significant at 0.05 level of significance.

Among participants, overall DHL increased by 3.01 score when frequency of reading health information about COVID-19 on the internet increased by one score (β = 3.01, 95% CI = 2.74 to 3.28). Whereas, overall DHL decreased by 2.01 score when confidence in the accuracy of the information on the internet regarding COVID-19 increased by one score (β = −2.01, 95% CI = −2.22 to −1.79) (Table 5).

**Table 5:** Linear relationship between overall DHL and health information-seeking behavior (*n* = 301).

**Health information-seeking behavior**	**β (95% CI)**	***p*** **-value**
How often do you read information about COVID-19 on the internet?	3.01 (2.74 to 3.28)	0.001^*^
I am confident with the accuracy of the information I read about COVID-19 on social media	−2.01 (−2.22 to −1.79)	0.001^*^

Linear regression was applied.

* Significant at 0.05 level of significance.

Multivariate linear regression revealed that health information-seeking behavior remained statistically associated with overall DHL even after adjusting for covariates like age, gender, marital status, educational status, employment status and household income. The adjusted R^2^ shows that independent variables can explain 60% of the variance in overall DHL (Table 6).

**Table 6:** Multivariate linear regression model for overall DHL and health information seeking behavior adjusted for covariates (*n* = 301).

**Health information-seeking behavior**	**β (95% CI)**	***p*** **-value**
How often do you read information about COVID-19 on the internet?	2.124 (1.73 to 2.52)	0.001^*^
I am confident with the accuracy of the information I read about COVID-19 on social media.	−0.846 (−1.13 to −0.56)	0.001^*^

Multivariate linear regression was applied.

* Significant at 0.05 level of significance.

Model adjusted for age, gender, marital status, educational status., employment status and household income.

## Discussion

The present study examines the impact of online health information-seeking behaviors on DHL related to COVID-19 among the B40 lower-income group in Selangor, Malaysia. The DHL increased with the frequency of reading information about COVID-19 on the internet and reduced with the reduced confidence about the accuracy of the COVID-19 information searched for.

It was elementary for participants to search for information on the internet (39.1%) compared with other components of DHL, such as adding self-generated content, evaluating reliability, or protecting privacy. This can lead to many individuals searching for and finding low-quality information that can lead to improper self-management of COVID-19 symptoms, as reported in other countries (38). There is also the risk of a breach of privacy to information of these individuals with the lowest socio-economic status in the country being targeted by internet scammers. Individuals in the B40 categories easily become pray to scammers because they are not used to using the internet and its associated tools making them have lower levels of DHL compared to other income groups. For example, previous researchers in other parts of the globe have identified lower levels of DHL among individuals in the B40 categories (39). To the extent that individuals in developed countries use digital health tools to monitor their health making digital platforms, user friendly for many individuals not in the B40 categories (40). This is mainly attributed to high-income and middle-income earners/countries having better access to technology and health facilities than the B40 lower-income group. This makes the better earners used to the internet and knowing the trusted sources of where to search for information.

In the analysis concerning participant characteristics and the levels of DHL, the widowed statistically had a higher mean of DHL content, relevance, reliability, and privacy than other marital statuses. This may be due to widows using platforms to seek support, or to inform others about their sorrows and worries, as a means of coping with the loss of a loved one (41–43). The constant use made their literary higher in most aspects of DHL, especially concerning COVID-19. However, the widow(er)s were least confident in the information obtained.

Study findings showed that DHL decreased with increasing level of education, a finding contradictory with previous studies (44, 45). This may be due to differences in the participants recruited in the previous studies, i.e., Adil et al. (45) university students that excluded community members and Flynn et al. (44) was conducted before the internet became popular among individuals with lower levels of education (44, 46). There are inconsistent findings around the extent of vaccine hesitancy by the level of education, suggesting that political variables are important confounders when considering education. For example, the government will often be responsible for the public health messages around COVID-19 vaccination through the Ministry of Health. Research from Ghana shows that if the individual voted for the opposition party, trust in the messaging is lower, with increased hesitancy (47). The delivery of public health messaging is important, and thus here, similar behavior may affect how people choose to search for and receive the required information. Also, the controversial finding with level of education and DHL may be due to use of a tool used to measure DHL that was not previously validated in similar a population; despite the good content and face validity.

The increase in DHL over the years may explain the higher DHL related to COVID-19 among unemployed individuals (46). Generally, many individuals are finding digital platforms more user friendly, with the migration to a digital era, and during the COVID-19 pandemic people explored the digital platforms for information and updates than any previous period. In addition, unemployed individuals may be exposed to more information online due to having adequate time spent online searching for employment. Here, participants earning a lower wage added increasing amounts of DHL content, whilst reading information about COVID-19 increased with age in the present study. This may be due to many older individuals being more concerned about the likely severity of illness and mortality in their populations and thus seeking out information on how best to protect themselves (48). Other demographics, for example, bereaved or widowed individuals, are potentially psychologically vulnerable to misinformation, so there is a fundamental importance to ensure that these groups can easily access appropriate health content.

Many individuals/groups with higher DHL were also reading more about COVID-19, but the more information they read, the lower their confidence in the information got. Individuals who get access to a lot of information find many contradictory findings, making them not confident of the information they read. They may be exposed to good and bad public health messaging but also see genuine uncertainties within the knowledge base, making it harder for an individual to make the best possible decisions. Due to the effect of the pandemic, such as emerging new variants, treatments, and vaccines (49), an increase in health information-seeking behaviors was associated with increased reading about COVID-19 information. Similar to other studies done during the pandemic, an increase in health information-seeking behaviors was associated with reduced confidence in the information obtained on social media (50–52). Social media has been the main source of spreading wrong information during the pandemic, especially by individuals who are against the vaccines and the lockdown protocols (53). Such misinformation on these social media platforms may also hinder the acceptance of good public health messaging.

This study has a few limitations. The first pertains to the use of convenience sampling and its cross-sectional nature. It cannot, therefore, be used to infer causality. Second, data were collected from participants' self-reports; thus, these may be subjected to socially desirable responses, and recall bias is common. Despite these limitations, the study data contribute to the understanding of the influence of DHL on health information-seeking behavior.

## Conclusion

The present study examines the impact of online health information-seeking behaviors on DHL related to COVID-19 among the B40 income group in Malaysia. An inverse relationship was found between confidence in the accuracy of the information on the internet regarding COVID-19 and DHL. It is important to support lower-income demographics to assist access to high-quality health information, including less educated, unemployed, and widowed populations in order to improve overall DHL.

Further research could replicate this study with other populations, and longitudinal studies could consider how temporal trends around health information-seeking behavior, for example, across the pandemic and also outside of times of public health emergencies. Authorities and health promotion teams can use the information here to consider pandemic strategies around health promotion in lower-income demographics in Malaysia.

## Data availability statement

The raw data supporting the conclusions of this article will be made available by the authors, without undue reservation.

## Ethics statement

The studies involving human participants were reviewed and approved by Ethics Committee of Management and Science University (Ethics Code: MSU-RMC-02/FR01/09/L1/085). The patients/participants provided their written informed consent to participate in this study.

## Author contributions

All authors made a significant contribution to the work reported, whether that is in the conception, study design, execution, acquisition of data, analysis and interpretation, or in all these areas, took part in drafting, revising or critically reviewing the article, gave final approval of the version to be published, have agreed on the journal to which the article has been submitted, and agree to be accountable for all aspects of the work.

## Funding

This work was supported by the Special Projects of the Central Government Guiding Local Science and Technology Development, China [No. 2021L3018]. The funder was not involved in study design, in the collection, analysis and interpretation of data, in the writing of the manuscript, nor in the decision to submit the manuscript for publication.

## Conflict of interest

The authors declare that the research was conducted in the absence of any commercial or financial relationships that could be construed as a potential conflict of interest.

## Publisher's note

All claims expressed in this article are solely those of the authors and do not necessarily represent those of their affiliated organizations, or those of the publisher, the editors and the reviewers. Any product that may be evaluated in this article, or claim that may be made by its manufacturer, is not guaranteed or endorsed by the publisher.
